# A synthesis of meta-analytic evidence of behavioral interventions to reduce HIV/STIs

**DOI:** 10.1007/s10865-016-9714-1

**Published:** 2016-01-30

**Authors:** Judith Covey, Harriet E. S. Rosenthal-Stott, Stephanie J. Howell

**Affiliations:** Department of Psychology, Wolfson Research Institute for Health and Wellbeing, Durham University, Queen’s Campus, Stockton-on-Tees, TS17 6BH UK; Department of Psychology, University of York, Heslington, York, YO10 5DD UK

**Keywords:** STI/HIV prevention, Intervention, Condom use, Systematic review, Meta-analysis, Meta-review, Intervention content, Mode of delivery, Communicator

## Abstract

To identify the mode of delivery, communicator, and content dimensions that make STI/HIV prevention interventions most successful at increasing condom use/protected sex or reducing STI/HIV incidence. A literature search for published meta-analyses of STI/HIV prevention interventions yielded 37 meta-analyses that had statistically tested the moderating effects of the dimensions. Significant and non-significant moderators from the coded dimensions were extracted from each meta-analysis. The most consistently significant moderators included matching the gender or ethnicity of the communicator to the intervention recipients, group targeting or tailoring of the intervention, use of a theory to underpin intervention design, providing factual information, presenting arguments designed to change attitudes, and providing condom skills and intrapersonal skills training. The absence of significant effects for intervention duration and expert delivery are also notable. The success of HIV/STI prevention interventions may be enhanced not only by providing skills training and information designed to change attitudes, but also by ensuring that the content is tailored to the target group and delivered by individuals of the same gender and ethnicity as the recipients.

**Electronic supplementary material:**

The online version of this article (doi:10.1007/s10865-016-9714-1) contains supplementary material, which is available to authorized users.

## Introduction

Since the 1980s and 1990s, numerous trials have been conducted to test the efficacy of behavioral interventions that aim to prevent sexually transmitted infections (STIs) and human immunodeficiency virus (HIV) by encouraging people to use condoms or reduce their number of sexual partners. In turn, in the last 10–15 years a large number of meta-analyses have been published. Some of these have focused on different target groups such as African Americans (Darbes et al., 2008), adolescents (Johnson et al., 2003), or men who have sex with men (MSM) (Johnson et al., 2005), or different types of interventions such as the use of computer-technology (Noar et al., 2009) or social media (Swanton et al., 2015). Despite their different foci, these meta-analyses often show positive pooled effect sizes for changes in condom use and other sexual risk behaviors. However, the effect sizes have been found to be significantly heterogeneous, which has led some researchers to explore which factors moderate intervention efficacy through stratified analysis and meta-regression techniques.

Given the growing numbers of meta-analyses that have conducted moderator analyses, researchers are now turning to systematically reviewing the meta-analytic studies themselves. Five such meta-reviews, or meta-syntheses, have been published in recent years. Each provide different insights into the moderators of intervention efficacy effect size (Johnson et al., 2014; Lorimer et al., 2013; Noar, 2008; Protogerou & Johnson, 2014; Vergidis & Falagas, 2009).

Four out of the five meta-reviews have focused their attention on meta-analyses of interventions targeted at specific groups such as MSM, adolescents, or specific ethnicities (Lorimer et al., 2013; Noar, 2008; Protogerou & Johnson, 2014; Vergidis & Falagas, 2009). A range of factors have been shown to be associated with larger intervention effects. Sessions delivered to single-ethnicity or single-gender groups were more efficacious than mixed ethnicity/gender sessions (Noar, 2008). For African Americans, greater efficacy was found for interventions that involved peer education, whereas for Latinos the effect was larger in interventions targeted at same sex groups (Vergidis & Falagas, 2009). Group and community-level interventions increased condom use and reduced unprotected anal intercourse in interventions delivered to MSM (Lorimer et al., 2013). The use of motivation enhancement skills training and use of theory was linked to efficacy in interventions targeted to adolescents (Protogerou & Johnson, 2014).

Unlike these four meta-reviews, Johnson et al. (2014) did not restrict their synthesis to prior meta-analyses focused on particular target groups. They focused instead on the 56 behavioral HIV prevention meta-analyses that had been included in a meta-synthesis of behavior change interventions conducted by Johnson et al., (2010). Two intervention content dimensions, skills training and motivational enhancement, were identified as being significantly associated with greater risk reduction behaviors in multiple meta-analyses. However, the synthesis lacked detail about the results found for all intervention content dimensions. In particular, their focus was on identifying only the significant moderators; the non-significant dimensions were not identified. This limits our ability to explore not only the reasons for lack of consensus in results between meta-analyses (i.e., why is a dimension a significant moderator in one meta-analysis but not another?), but also to identify dimensions that never, or rarely, produce significant effects (i.e., which dimensions do not make a difference to intervention effectiveness?)

This limitation is addressed in the meta-review reported in this paper, in which we present a comprehensive and detailed synthesis of previous meta-analyses that have tested the significance of intervention dimensions. The intervention dimensions selected for analysis are listed and defined in Table 1 and include mode of delivery dimensions (e.g., number of sessions, group delivery) and communicator dimensions (e.g., matched ethnicity, expert delivery), as well as the content dimensions (e.g., individual tailoring, condom skills training) analyzed by Johnson et al. (2014). Also, unlike the meta-reviews conducted by Lorimer et al. (2013), Noar (2008), Protogerou and Johnson (2014), and Vergidis and Falagas (2009) we did not restrict our analysis to meta-analyses that had focused on particular target groups like MSM, adolescents, or specific ethnicities.

**Table 1:** Intervention characteristic dimensions

*Mode of delivery dimensions*	
Duration	Total duration of the intervention
Session number	Total number of sessions over which the intervention was delivered
School setting	Delivered in a school, classroom or educational setting
Clinic setting	Delivered in a clinic or health care setting
Community setting	Delivered in a community setting
Group delivery	Delivered in a group setting rather than to individuals
*Communicator dimensions*	
Peer delivery	Delivered by a peer or involved peer group discussion/education
Expert delivery	Delivered by an expert (including health care providers/counselors)
Matched ethnicity	Delivered by a person of the same ethnicity as the recipient
Matched gender	Delivered by a person of the same gender as the recipient
Similar age	Delivered by a person of a similar age as the recipient
*Content dimensions*	
Group targeting/tailoring	Intervention targeted at a specific group or intervention tailored to enhance its applicability and acceptability to a particular group. Groups may be based on characteristics such as gender, ethnicity, culture, sexuality or age.
Individual tailoring	The materials used for the intervention were tailored to each individual recipient
Formative research	The intervention was underpinned by previously conducted (formative) research
Theory-based	The intervention was underpinned by a theory of health behavior
Information	Provided information about the mechanisms of HIV, STI/HIV transmission or disease prevention methods (e.g., condom use)
Motivational enhancement	Included a motivational enhancement component or training
Threat/fear induction	Included threat/fear-inducing arguments or addressed perceptions or risk
Attitudinal arguments	The intervention included arguments aimed to change people’s attitudes towards risky sexual behavior and using condoms
Normative arguments	Included normative arguments which addressed social norms towards safer sex and/or peer influence
Address barriers	Addressed barriers to condom use
Address self-efficacy	Addressed self-efficacy beliefs about safer sex and/or protective behavior
Behavioral skills arguments	Included behavioral skills arguments
Skills (mixed)	Included various types of skills training or included skills training without specifying the exact skills that were addressed
Condom skills	Included condom use skills training
Intrapersonal skills	Included intrapersonal skills training not restricted to condom use (including self-management, self-control, decision making)
Interpersonal skills	Included interpersonal skills training (including communication/condom use negotiation)

### Objectives

The aim of this meta-review was to synthesize the existing meta-analytic evidence on the outcomes of behavioral interventions that aim to reduce the risk of STIs or HIV by increasing condom use or reducing unprotected sex. Our primary objective was to identify which types of interventions previous meta-analyses have found to be associated with larger intervention effects. We considered a broad range of intervention characteristics shown and defined in Table 1, which included format of delivery dimensions (e.g., number of sessions, group delivery), communicator dimensions (e.g., matched ethnicity, expert delivery), and content dimensions (e.g., individual tailoring, condom skills training).

## Methods

### Eligibility criteria

To qualify for inclusion, the meta-analysis must have: (1) been published in a peer-reviewed journal since 2000; and (2) reported moderator analysis with significance testing for at least one of the intervention features (shown in Table 1) on sexual risk behavior (i.e., measures of condom use or unprotected sex) or STI/HIV incidence rates. Meta-analyses were excluded if they: (1) focused only on interventions that aimed to prevent pregnancy without also addressing the prevention of STIs or HIV; (2) focused only on interventions that aimed to prevent HIV/STI transmission from people living with HIV (including mother–child transmission of HIV), or were concerned only with evaluating the outcomes of STI screening, HIV counselling/testing or HPV vaccination; (3) focused only on abstinence education interventions aimed at reducing sexual activity rather than encouraging condom use/protection; or (4) only reported moderator analysis on effect sizes based on sexual activity measures such as number of sexual partners or frequency of sexual activity.

### Information sources, search strategy and study selection

**Fig. 1:**
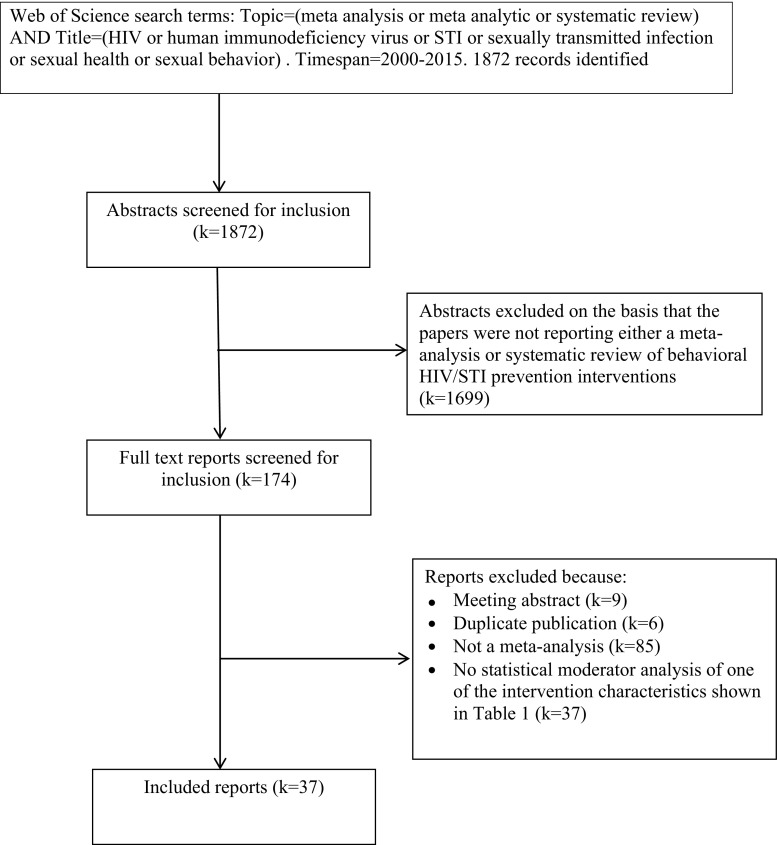
PRISMA flowchart of study inclusion and exclusion

The Web of Science (formerly Web of Knowledge) database was searched on May 7 2015. In addition to the Web of Science Core Collection [Social Sciences Citation Index (SSCI), Science Citation Index Expanded (SCI-EXPANDED)], this database includes access to the Cochrane Database of Systematic Reviews, Current Contents Connect, and MEDLINE. The search terms used are shown in Fig. 1, which also shows the PRISMA flowchart of study inclusion and reasons for exclusion (Moher et al., 2009).

JC and HR-S independently screened the titles and abstracts of the papers identified from the search. Potentially eligible papers were short-listed for full-text review if the title or abstract indicated that the paper was reporting either a meta-analysis or systematic review of STI/HIV prevention interventions. The full-text articles were then reviewed by both JC and HR-S and only papers that met the eligibility criteria were included in the synthesis.

### Data extraction and analysis

We extracted the following information from each meta-analysis: (1) authors and report date; (2) type of STI/HIV interventions included in the meta-analysis; (3) target group(s) included or excluded from the meta-analysis (including country of residence restrictions); (4) latest year included in the search period; and (5) details of the moderator analysis reported for the intervention characteristics shown in Table 1. We recorded: (1) the number of studies (k) on which the moderator analysis was based; (2) whether the moderator analysis was conducted on a univariate or multivariate basis; (3) whether the researchers had used conservative Bonferroni corrected significance levels for multiple comparisons; and (4) whether the moderator effect was significantly positive (+), negative (−), or not significant (ns). Data extraction was conducted by JC and checked by either SH or HR-S. Fewer than 6 differences in coding were identified across all meta-analyses and these were resolved by discussion.

## Results

As shown in Fig. 1, 37 meta-analyses were included in this meta-review. Table 2 shows the data extracted from each study. The meta-analyses varied in terms of how inclusive they were with some focusing on specific types of populations such as adolescents (Chin et al., 2012; Johnson et al., 2003; Johnson et al., 2011; Mullen et al., 2002), STI clinic patients (Crepaz et al., 2007; Scott-Sheldon et al., 2010), African Americans (Crepaz et al., 2007, 2009; Darbes et al., 2008; Henny et al., 2012; Johnson et al., 2009; Reid et al., 2014), Hispanics (Crepaz et al., 2007; Herbst et al., 2007), MSM (Herbst et al., 2005; Higa et al., 2013; Johnson et al., 2005), heterosexuals (Henny et al., 2012; LaCroix et al., 2013; Neumann et al., 2002; Tyson et al., 2014), women only (Crepaz et al., 2009; Lennon et al., 2012), men only (Henny et al., 2012), or drug users (Meader et al., 2013; Prendergast et al., 2001). Beyond the interventions tested on North American populations, which were included in most of the meta-analyses, others were restricted to particular countries like South Africa (Scott-Sheldon et al., 2013) and China (Liu et al., 2014; Xiao et al., 2012; Zheng & Zheng, 2012), or Asian countries (Tan et al., 2012).

**Table 2:** Tests of moderator effects on condom use/unprotected sex and STI/HIV incidence effect sizes in 37 meta-analyses of HIV prevention interventions

Authors (year)	Types of interventions/populations included and excluded (latest search year)	Positive (+), negative (−) and non-significant (ns) effects Moderators found to be significant in multivariate tests are *italicized*	
	Moderator analysis (k)	Condom use/unprotected sex	STI/HIV incidence
Albarracin et al. (2005)	HIV prevention interventions. Studies must include a pre-test and post-test. (2003) Univariate analyses were conducted for all moderators apart from the use of formative research. For some moderators analyses were conducted separately for active (k = 123) and passive (k = 77) interventions. The results were the same for both types of interventions unless otherwise indicated	(+) Information (active), Gender group targeting/tailoring, Attitudinal arguments, Behavioral skills arguments, Intrapersonal skills, Theory-based (−) Threat/fear induction (active), Normative arguments (active), Interpersonal skills, Formative research (ns) Duration, School setting, Clinic setting, Community setting, Group delivery, Ethnic group targeting/tailoring, Information (passive), Threat/fear induction (passive), Normative arguments (passive), Condom skills	Not tested
Albarracin et al. (2003)	Condom use communications (verbal, written or visual). Excluded studies in which recipients engaged in behaviors (e.g., role playing). Studies must include a pre-test and post-test. (1998). Multivariate analyses were only conducted for communication arguments (e.g., attitudinal arguments/behavioral skills arguments) controlling for methodological features (k = 40)	(+) Formative research, *Attitudinal arguments*, *Behavioral skills arguments* (−) Information, School setting (ns) Duration, Threat/fear induction, Normative arguments	Not tested
Albarracin et al. (2008)	HIV prevention interventions with focus on condom-use. Studies must include a pre-test and post-test and provide information about the percent of Latinos in the sample. (2005) Multivariate moderator analysis was conducted separately on studies according to whether they included a high % (k = 33) or low % of Latinos (k = 317). The results were the same for both groups of studies unless otherwise indicated	(+) *Clinic setting (Low* *% Latino), Group delivery (Low* *% Latino), Expert delivery (Low* *% Latino), Matched ethnicity (Low* *% Latino), Matched gender (Low* *% Latino), Similar age (Low* *% Latino), Information (Low* *% Latino), Threat/fear induction (High* *% Latino), Attitudinal argument (Low* *% Latino), Behavioral skills arguments (Low* *% Latino), Condom skills (Low* *% Latino), Intrapersonal skills (Low* *% Latino)* (−) *Clinic setting (High* *% Latino), Community setting (Low* *% Latino), Threat/fear induction (Low* *% Latino), Attitudinal arguments (High* *% Latino), Normative arguments, Behavioral skills arguments (High* *% Latino), Condom skills (High* *% Latino), Intrapersonal skills (High* *% Latino), Interpersonal skills (High* *% Latino)* (ns) Community setting (High % Latino), Group delivery (High % Latino), Expert delivery (High % Latino), Matched ethnicity (High % Latino), Matched gender (High % Latino), Similar age (High % Latino), Interpersonal skills (Low % Latino)	Not tested
Chin et al. (2012)	Group-based HIV/STI and comprehensive risk reduction interventions^a^ conducted on adolescents (10–19 years) in school or community settings. (2007) This meta-analysis reported effect sizes for a range of sexual risk behaviors including condom use (k = 48), unprotected sexual activity (k = 29) and STI incidence (k = 8). Univariate moderator analysis was conducted on all of these measures with no significant moderator effects reported	(+) None reported (−) None reported (ns) Duration, School setting, Community setting, Peer delivery, Group targeting/tailoring	(+) None reported (−) None reported (ns) Duration, School setting, Community setting, Peer delivery, Group targeting/tailoring,
Crepaz et al. (2007)	Behavioral STI/HIV prevention interventions conducted on STI clinic patients with at least 50 % Black/Hispanics USA only. (2004) Univariate moderator analysis on condom use/unprotected sex (k = 14) and STI incidence (k = 13)	(+) Matched ethnicity (−) Expert delivery (ns) Duration, Session number, Clinic setting, Group delivery, Group targeting/tailoring, Formative research, Threat/fear induction, Attitudinal arguments, Address self-efficacy, Condom skills, Intrapersonal skills, Interpersonal skills	(+) Matched ethnicity, Theory-based (−) Threat/fear induction, Attitudinal arguments (ns) Duration, Session number, Clinic setting, Expert delivery, Group targeting/tailoring, Formative research, Address self-efficacy, Condom skills, Intrapersonal skills, Interpersonal skills
Crepaz et al. (2009)	Behavioral STI/HIV prevention interventions conducted on female populations with at least 50 % African Americans USA only. (2007) Univariate moderator analysis on condom use/unprotected sex (k = 33) and STI incidence (k = 17)	(+) Matched gender, Group targeting/tailoring, Address self-efficacy, Condom skills (−) None reported (ns) Duration, Session number, Clinic setting, Community setting, Group delivery, Peer delivery, Matched ethnicity, Formative research, Motivation enhancement, Normative arguments	(+) Duration, Peer delivery, Formative research, Address self-efficacy (−) None reported (ns) Session number, Clinic setting, Community setting, Group delivery, Group targeting/tailoring, Motivation enhancement, Normative arguments, Condom skills
Darbes et al. (2008)	Individual-level and group-level interventions conducted on heterosexual populations with at least 80 % African Americans USA only. (2005) Univariate moderator analysis on condom use/unprotected sex (k = 35) and STI incidence (k = 10)	(+) Peer delivery, Normative arguments (−) None reported (ns) Duration, Session number, Clinic setting, Community setting, Group delivery, Matched ethnicity, Group targeting/tailoring, Theory-based, Motivation enhancement, Attitudinal arguments, Address self-efficacy, Skills (mixed)	(+) None reported (−) None reported (ns) Duration, Session number, Clinic setting, Community setting, Group delivery, Peer delivery, Matched ethnicity, Group tailoring/targeting, Theory-based, Motivation enhancement, Attitudinal arguments, Normative arguments, Address self-efficacy, Skills mixed
Durantini et al. (2006)	HIV prevention interventions with focus on condom use. Studies must include a pre-test and post-test and provide information about the interventionist. (2003) For some moderators analyses were reported separately according to whether the recipients were predominantly male or female, African or European, and <21 or >21. The results were the same for all groups unless otherwise indicated. Univariate analyses were conducted for all moderators apart from the use of formative research. (k = 166)	(+) Clinic setting, Group delivery, Expert delivery (African, >21), Matched ethnicity (African), Matched gender (female), Similar age (European, <21), Gender group targeting/tailoring, Information, Theory-based, Behavioral skills arguments, Condom skills, Interpersonal skills, Intrapersonal skills (−) School setting, Community setting, Formative research, Threat/fear induction, Attitudinal arguments, Normative arguments (ns) Duration, Expert delivery (European;, <21), Matched ethnicity (European), Matched gender (male), Similar age (African, >21), Ethnic group targeting/tailoring	Not tested
Earl and Albarracin (2007)	HIV prevention interventions with focus on condom use. Studies must include a pre-test and post-test and include measures of change at both an immediate and delayed follow-up. (2005) Multivariate moderator analysis (k = 180)	(+) None reported (−) *Threat/fear induction* (ns) None reported	Not tested
Eaton et al. (2012)	Single-session behavioral interventions for STI prevention. (2011) Univariate moderator analysis on STI incidence (k = 29)	Not tested	(+) Duration (−) None reported (ns) None reported
Henny et al. (2012)	HIV prevention interventions conducted on male populations with at least 50 % African Americans and at least 50 % heterosexuals -community-level interventions excluded USA only. (2008) Moderators identified as significant in a univariate analysis were then tested in a multivariate model (k = 40)	(+) Matched gender (−) None reported (ns) Duration, Session number, Matched ethnicity, Information, Group targeting/tailoring, Formative research, Motivation enhancement, Attitudinal arguments, Normative arguments, Condom skills, Interpersonal skills, Intrapersonal skills	Not tested
Herbst et al. (2007)	HIV/STI behavioral interventions conducted on populations with at least 50 % Hispanics USA or Puerto Rico only. (2005) Univariate moderator analysis (k = 19)	(+) Session number, Normative arguments, Address barriers (−) Peer delivery (ns) Clinic setting, Community setting, Group delivery, Matched ethnicity, Formative research, Theory-based, Address self-efficacy, Condom skills, Interpersonal skills, Intrapersonal skills	Not tested
Herbst et al. (2005)	HIV/STI behavioral interventions conducted on populations with at least 85 % MSM (2003) Univariate moderator analysis (k = 19)	(+) Theory-based (−) None reported (ns) None reported	Not tested
Higa et al. (2013)	HIV prevention interventions specifically designed for MSM USA only (2011) Univariate analysis^b^	(+) Peer delivery, Interpersonal skills (−) None reported (ns) Duration, Session number, Group delivery	Not tested
Huedo-Medina et al. (2010)	HIV/AIDS behavioral interventions involving face-to-face interactions Latin America and Caribbean only (2009) Moderators identified as significant in a univariate analysis were then tested in a multivariate model (k = 32). Bonferroni corrected significance values used (*p* = .01)	(+) None reported (−) School setting, Group delivery, Group targeting/tailoring (ns) Duration, Individual tailoring, Address barriers, Interpersonal skills	Not tested
Johnson et al. (2003)	HIV sexual risk-reduction interventions in pre-college adolescents—excluded pamphlet studies (2000) Moderators identified as significant in a univariate analysis were then tested in a multivariate model (k = 42)	(+) Theory-based, *Condom skills* (−) None reported (ns) School setting, Group delivery, Peer delivery, Matched ethnicity, Matched gender, Individual tailoring	Not tested
Johnson et al. (2011)	HIV sexual risk-reduction interventions in pre-University adolescents 11–19 years—excluded pamphlet studies (2008) Moderators identified as significant in a univariate analysis were then tested in a multivariate model (k = 91). Only motivation enhancement and condom skills were significant in the multivariate model	(+) *Motivation enhancement*, *Condom skills* (−) None reported (ns) Session number, Individual tailoring, Interpersonal skills	Not tested
Johnson et al. (2009)	HIV risk reduction interventions conducted in populations with at least 50 % African Americans USA only (2006) Moderator analysis was conducted separately for condom use in the short-term (k = 68), intermediate (k = 59) and long-term (k = 28). The results were the same at all follow-ups unless otherwise indicated. Moderators identified as significant in a univariate analysis were then tested in a multivariate model. Only duration (intermediate, long-term) and intrapersonal skills (short-term) were significant in the multivariate model	(+) *Duration (intermediate, long*-*term)*, Individual tailoring (long-term), Interpersonal skills (intermediate, long-term), *Intrapersonal skills (short*-*term)* (−) None reported (ns) Duration (short-term), Individual tailoring (short-term), Interpersonal skills (short-term), Intrapersonal skills (intermediate, long-term)	Not tested
Johnson et al. (2005)	HIV prevention interventions in populations with a high MSM percentage (2005) Stepwise regression used to identify moderators associated with the most favorable effect sizes for individual (k = 18), community (k = 10) and group level (k = 10) interventions.	(+) *Threat/fear induction (individual, group)*, *Intrapersonal skill (community)* (−) None reported (ns) Threat/fear induction (community), Intrapersonal skills (individual, group)	Not tested
LaCroix et al. (2013)	Heterosexual couple-based HIV prevention interventions on condom use (2013) Univariate moderator analysis (k = 28)	(+) Condom skills (−) Group delivery (ns) Duration	Not tested
LaCroix et al. (2014)	Mass media HIV prevention interventions targeted on youth/general population in natural settings—excluded interventions on high-risk groups (2013) Univariate moderator analysis (k = 58)	(+) Duration, Group targeting/tailoring (−) None reported (ns) None reported	Not tested
Lennon et al. (2012)	Face-to-face HIV prevention interventions that measured depression and reported separate results for women (2010) Univariate moderator analysis (k = 23)	(+) Information (−) None reported (ns) None reported	Not tested
Liu et al. (2014)	HIV prevention interventions in floating^c^ populations in mainland China-excluded high risk groups such as MSM, sex workers and drug users excluded (2012) Moderators identified as significant in a univariate analysis were then tested in a multivariate model	(+) None reported (−) None reported (ns) Peer delivery	Not tested
Meader et al. (2013)	Multisession psychosocial interventions on drug users compared against educational interventions (2000) Univariate moderator analysis (k = 46)	(+) None reported (−) None reported (ns) Clinic setting, Motivation enhancement, Condom skills	Not tested
Mullen et al. (2002)	HIV behavioral and social interventions on adolescents (13–19 years) conducted in school and out of school settings USA only (1998) Univariate moderator analysis (k = 16). Although Bonferroni corrected significance levels were used (*p* = .004) all the *p* values for the non-significant effects reported here were greater than the uncorrected significance level of *p* = .05 that was used in the majority of meta-analyses reported in this paper	(+) None reported (−) None reported (ns) Session number, School setting, Threat/fear induction, Interpersonal skills, Intrapersonal skills	Not tested
Neumann et al. (2002)	HIV behavioral and social interventions on heterosexuals over 21 years USA only (1996) Univariate moderator analysis (k = 10)	(+) Group delivery (−) None reported (ns) Clinic setting, Skills mixed	Not tested
Noar et al. (2009)	Computer-technology based HIV prevention interventions (2008) Univariate moderator analysis (k = 12)	(+) Session number, Individual tailoring (−) None reported (ns) Theory-based, Skills mixed	Not tested
Prendergast et al. (2001)	HIV risk reduction interventions on drug abuse treatment clients USA and Canada only (1998) Univariate moderator analysis (k = 14)	(+) Peer delivery, Intrapersonal skills (−) None reported (ns) Duration, Skills mixed	Not tested
Reid et al. (2014)	HIV prevention interventions on African Americans USA only (2006) Univariate moderator analysis (k = 99) conducted in communities where Whites had either a negative or positive attitude towards African Americans (k = 99)	(+) Group targeting/tailoring (Whites negative attitude) (−) None reported (ns) Group targeting/tailoring (Whites positive attitude)	Not tested
Scott-Sheldon et al. (2010)	Individual or group-level behavioral interventions on STI clinic patients USA only (2009) Moderator analysis was conducted separately for condom use/STI incidence in the short-term (k = 31/k = 8), intermediate (k = 26/k = 21) and long-term (k = 13/k = 5). The results were the same at all follow-ups unless otherwise indicated. Moderators identified as significant in a univariate analysis were then tested in a multivariate model	(+) Duration, *Group targeting/tailoring* (−) Individual tailoring (intermediate, long-term) (ns) Individual tailoring (short-term), Motivation enhancement, Skills mixed	(+) *Motivation enhancement (intermediate)* (−) Duration (short-term), Individual tailoring (short-term), Motivation enhancement (short-term), Skills mixed (short-term) (ns) Duration (intermediate, long term), Group targeting/tailoring (intermediate, long term), Individual tailoring (intermediate, long-term), Motivation enhancement (long-term), Skills mixed (intermediate, long-term)
Scott-Sheldon et al. (2011)	STI/HIV behavioral interventions-excluded mass media/structural (2010) Moderator analysis conducted on condom use (k = 76), STI incidence (k = 62) and HIV incidence (k = 13), Moderators identified as significant in a univariate analysis were then tested in a multivariate model	(+) Cultural group targeting/tailoring, *Address barriers* (−) Intrapersonal skills (ns) Duration, Matched ethnicity, Matched gender, Gender group targeting/tailoring, Individual tailoring, Motivation enhancement, Condom skills, Interpersonal skills	(+) Gender targeting/tailoring (HIV incidence), Motivation enhancement (HIV incidence), Condom skills (HIV incidence) (−) Intrapersonal skills (STI incidence) (ns) Duration, Matched ethnicity (STI incidence), Matched gender, Cultural targeting/tailoring (STI incidence), Gender targeting/tailoring (STI incidence), Individual tailoring, Motivation enhancement (STI incidence), Address barriers, Condom skills (STI incidence), Intrapersonal skills (HIV incidence), Interpersonal skills (STI incidence)
Scott-Sheldon et al. (2013)	Behavioral interventions to reduce sexual risk behaviors and the incidence of STIs in South African youth 9–26 years (2013) Moderators identified as significant in a univariate analysis were then tested in a multivariate model (k = 10). Although Bonferroni corrected significance levels were used (*p* = .005) the *p* value for the non-significant effect of normative arguments reported here was greater than the uncorrected significance level of *p* = .05 that was used in the majority of meta-analyses reported in this paper	(+) Expert delivery, Condom skills (−) Duration, Session number (ns) Normative arguments	Not tested
Swanton et al. (2015)	New-media-based sexual health interventions—e.g., social networking sites, smart phone apps (2014) Univariate moderator analysis (k = 12)	(+) None reported (−) None reported (ns) Duration	Not tested
Tan et al. (2012)	HIV prevention interventions conducted in Asia (2010) Univariate moderator analysis of condom use (k = 52) and STI/HIV incidence (k = 20). Moderators identified as significant in a univariate analysis were then tested in a multivariate model	(+) Group delivery, Motivation enhancement, Interpersonal skills (−) Duration, Individual tailoring (ns) Threat/fear induction, Attitudinal arguments, Condom skills	(+) Threat/fear induction (−) None reported (ns) Duration, Group delivery, Individual tailoring, Motivation enhancement, Attitudinal arguments, Condom skills, Interpersonal skills
Tyson et al. (2014)	STI/HIV prevention interventions on heterosexuals informed by the Theory of Planned Behavior (2013) Moderators identified as significant in a univariate analysis were then tested in a multivariate model (k = 34)	(+) Attitudinal arguments (−) None reported (ns) Information, Motivation enhancement, Normative arguments, Address barriers, Skills mixed	Not tested
Xiao et al. (2012)	HIV/sexual risk reduction interventions conducted in China (2011) Univariate moderator analysis (k = 25)	(+) Peer delivery, Formative research (−) Expert delivery (ns) Theory-based	Not tested
Zheng and Zheng (2012)	HIV prevention interventions conducted on MSM in China (2011) Univariate moderator analysis was conducted separated for condom use at the most recent intercourse (k = 16) and within the last six months (k = 16). The results were the same for both measures unless indicated	(+) None reported (−) Peer delivery. Individual tailoring (6 months) (ns) Individual tailoring (most recent)	Not tested

^a^This meta-analysis also examined the effects of group-based abstinence education interventions the analysis of which is not included in this meta-review because these types of interventions are not aimed at reducing unprotected sex/encouraging condom use. However it is worth noting that none of the tested moderators were significant for either type of intervention

^b^This review adopted a different approach to examining the role of intervention characteristics. Studies were coded according to the extent to which they met certain efficacy criteria (including whether the study had shown a significant positive intervention effect on a relevant behavioral or biological outcome). The intervention characteristics of effective interventions (EBIs) versus non-effective interventions (non-EBIs) were compared using Fisher’s exact test and non-parametric independent samples median tests

^c^Floating refers to Chinese citizens who live in an area different from the place where their household is registered in the “hukou” system

Some reviews also placed restrictions on the types of interventions that were included. Restrictions included excluding interventions where recipients engaged in behaviors like role playing or condom-use skills (Albarracin et al., 2003), pamphlet studies (Johnson et al., 2003; Johnson et al., 2011), or mass-media interventions (Scott-Sheldon et al., 2011). Others restricted themselves to interventions that were group-based (Chin et al., 2012), multi-session (Meader et al., 2013), single session (Eaton et al., 2012), face-to-face (Huedo-Medina et al., 2010; Lennon et al., 2012), used computer-technology (Noar et al., 2009), used new media (Swanton et al., 2015), or were informed by the Theory of Planned Behavior (Tyson et al., 2014).

The final types of restrictions were concerned with the study design or information provided in the intervention reports. Some meta-analyses only included studies that comprised both a pre-test and post-test (Albarracin et al., 2005; Albarracin et al., 2003; Albarracin et al., 2008; Durantini et al., 2006; Earl & Albarracin, 2007), or where information was provided about the interventionist (Durantini et al., 2006) or percentage of Latinos in the sample (Albarracin et al., 2008), or where depression measures were obtained and separate results were provided for women (Lennon et al., 2012).

Although these restrictions reduce the overlap between the meta-analyses included in this meta-review, several of the meta-analyses share the same intervention studies. For example, the analyses reported by Durantini et al. (2006) and Earl and Albarracin (2007) were both based on a sub-set of papers reviewed by Albarracin et al. (2005). All of the studies included in Johnson et al. (2003) were included in the later meta-analysis reported in Johnson et al. (2011), and Reid et al. (2014) report a secondary analysis of studies included in Johnson et al. (2009). The overlap is particularly important to consider when synthesizing and interpreting the results of the moderator analyses.

### Moderator analysis

Table 2 shows the results of the moderator tests conducted on the effect sizes for each meta-analysis and the overall numbers of significant and non-significant effects are summarized in Table 3. Some dimensions were tested as moderators more often than others. Frequently tested dimensions include duration, group targeting/tailoring, and skills training (condom, intrapersonal or interpersonal).

**Table 3:** Number of significant and non-significant moderator effects for the mode of delivery, communicator and content dimensions^a^

	Condom use/unprotected sex			STI/HIV incidence		
	(+)	(−)	ns (k < 20, B)^b^	(+)	(−)	ns (k < 20, B)^b^
*Mode of delivery dimensions*						
Duration	6	2	15 (4, 1)	2	1	8 (4, 0)
Session number	2	1	7 (3, 0)	0	0	3 (3, 0)
School setting	0	3	4 (1, 0)	0	0	1 (1, 0)
Clinic setting	2	1	7 (3, 0)	0	0	3 (3, 0)
Community setting	0	2	6 (1, 0)	0	0	3 (3, 0)
Group delivery	4	2	8 (3, 0)	0	0	3 (2, 0)
*Communicator dimensions*						
Peer delivery	4	3	4 (1, 0)	1	0	2 (2, 0)
Expert delivery	6	2	3 (0, 0)	0	0	1 (1, 0)
Matched ethnicity	6	1	7 (1, 0)	1	0	3 (2, 0)
Matched gender	8	0	4 (0, 0)	0	0	3 (2, 0)
Similar age	3	0	5 (0, 0)	–	–	–
*Content dimensions*						
Group targeting/tailoring	9	1	9 (2, 0)	1	0	8 (4, 0)
Individual tailoring	3	4	7 (1, 1)	0	0	5 (1, 0)
Formative research	2	2	4 (2, 0)	1	0	1 (1, 0)
Theory-based	4	0	4 (2, 0)	1	0	1 (1, 0)
Information	4	1	3 (0, 0)	–	–	–
Motivational enhancement	2	0	9 (1, 0)	2	1	5 (2, 0)
Threat/fear induction	3	4	6 (3, 1)	1	0	1 (1, 0)
Attitudinal arguments	5	2	4 (1, 1)	0	0	3 (2, 0)
Normative arguments	2	4	6 (1, 0)	0	0	2 (2, 0)
Address barriers	2	0	2 (0, 1)	0	0	2 (1, 0)
Address self-efficacy	1	0	3 (2, 0)	1	0	2 (2, 0)
Behavioral skills arguments	5	1	0 (0, 0)	–	–	–
Skills (mixed)	0	0	8 (4, 0)	0	1	3 (1, 0)
Condom skills	7	1	7 (2, 1)	1	0	4 (2, 0)
Intrapersonal skills	7	2	7 (2, 0)	0	1	2 (1, 0)
Interpersonal skills	5	2	9 (3, 1)	1	0	3 (1, 0)

^a^For further details of the meta-analyses that produced the significant and non-significant effects for each moderator see Online Resource 1 (condom use/unprotected sex) and Online Resource 2 (STI/HIV incidence)

^b^Number of non-significant effects that were based on reduced power factors—k < 20 or Bonferroni corrected significance levels

Although the numbers shown in Table 3 provide a snapshot of which dimensions were most and least likely to produce significant effects, the numbers need to be treated with caution for a couple of reasons. Firstly, significant effects were more likely to be produced in meta-analyses with larger numbers of studies—the 123 significant effects found for the condom use/unprotected sex effect sizes came from tests conducted on an average of 100 studies [M(95 % CI) = 100 (82–118), Mdn = 40, SD = 105, n = 123] whereas the 145 non-significant effects came from tests conducted on an average of 45 studies [M(95 %CI) = 45 (37–53), Mdn = 34, SD = 50, n = 145]. Secondly, the effects are not independent of each other. As well as meta-analyses sharing the same intervention studies, some meta-analyses tested moderator effects for multiple related outcomes, for example condom use in the short, intermediate and long-term (Johnson et al., 2009), or condom use at most recent sexual intercourse and within the last 6 months (Zheng & Zheng, 2012). It is therefore important to consider not only the numbers of significant and non-significant effects, but also the sources of the effects. We therefore examined whether there are features of the meta-analyses that differentiate the significant effects from the non-significant effects. Although this information can be extracted from Table 2, listing the findings for each dimension facilitates this analysis (see Online Resource 1 (condom use/unprotected sex) and Online Resource 2 (STI/HIV incidence). These Online Resources also report the effect sizes for the significant moderators when they were reported by the original meta-analyses. This provides a sense of the magnitude of the effects observed.

### Mode of delivery dimensions

With regard to mode of delivery dimensions, there is limited evidence that interventions of longer duration or consisting of more sessions are more efficacious. The majority of effects for duration were not significant and the 6 positive effects found for condom use/unprotected sex were obtained from 3 meta-analyses, 2 of which tested the effects of the moderator at 3 condom use follow-ups (Johnson et al., 2009; LaCroix et al., 2014; Scott-Sheldon et al., 2010). There is also no obvious distinction between the target groups or types of interventions included in these meta-analyses compared to those that produced non-significant effects.

Three out of 7 meta-analyses found interventions delivered in a school, classroom or educational setting were less effective at reducing sexual risk behaviors with small effect sizes (r = −.32, β = −.23, β = −.33) (Albarracin et al., 2003; Durantini et al., 2006; Huedo-Medina et al., 2010). However, since none of these three meta-analyses were restricted to interventions conducted on school- or college-aged populations the effect of this moderator might reflect lower efficacy of interventions in recipients of this age-range rather than the location of the intervention itself. This interpretation is supported by the fact that 3 of the 4 meta-analyses that produced non-significant effects of school setting had restricted their populations to adolescents (Chin et al., 2012; Johnson et al., 2003; Mullen et al., 2002). There is therefore little evidence that the setting (whether school, clinic or community) in which an intervention is delivered makes any difference to its effectiveness.

The effects of delivering an intervention in groups were also inconclusive. The 4 meta-analyses that demonstrated positive effects on condom use/unprotected sex for this moderator (Albarracin et al., 2008; Durantini et al., 2006; Neumann et al., 2002; Tan et al., 2012) do not appear to share any distinguishing features from the 10 that demonstrated negative or non-significant effects.

### Communicator dimensions

Turning to the communicator dimensions, the effects of peer and expert delivery are somewhat mixed. It might be worth noting that the 2 meta-analyses that produced the 3 significant negative effects on condom use/unprotected sex for peer delivery were based on populations that included a high percent of MSM (Herbst et al., 2007; Zheng & Zheng, 2012). However, the idea that peer delivery is less effective in MSM populations is weakened by the finding that 1 of the 4 meta-analyses that produced significant positive effects was also based on an analysis of interventions designed for MSM (Higa et al., 2013). There were no observable distinctions between the meta-analyses that showed positive or negative effects of expert delivery.

Matching the person delivering the intervention according to the ethnicity, gender or age of the recipient had positive effects on intervention effectiveness in the majority of tests on condom use/unprotected sex. Matching gender produced most of the significant positive effects, although the effects were quite small. As shown in Online Resource 1, Cohen’s d effect sizes were between .14 and .38 larger when the facilitator’s gender was matched to the recipient. Although the positive significant effects for matching ethnicity and age were of a similar magnitude, they were outweighed by non-significant or negative effects. However, the non-significant effects were obtained from meta-analyses with much smaller numbers of studies—6 out of the 7 non-significant effects came from meta-analyses with fewer than 50 studies, whereas 5 out of the 6 significant positive effects came from two meta-analyses with over 200 studies (Albarracin et al., 2008; Durantini et al., 2006).

### Content dimensions

The effects of group targeting/tailoring, where interventions were targeted at a specific group or tailored to enhance their applicability or acceptability to a particular group, were more likely to be positive than the effects of individual tailoring where materials used for the intervention were tailored to each individual recipient. However, there were no easily observable differentiating features between the meta-analyses that showed positive effects of group targeting/tailoring and those that showed non-significant effects. However, 2 of the 3 meta-analyses that found individual tailoring to have negative effects on condom use/unprotected sex were based on interventions conducted in Asia and China (Tan et al., 2012; Zheng & Zheng, 2012).

Conducting formative research had mixed effects. Although effects on condom use/unprotected sex were positive in 2 meta-analyses, they were negative in 2. However, these negative effects were small (β = −.12, β = −.08) and not significant when all methodological and population predictors were simultaneously entered into the analysis (Albarracin et al., 2005; Durantini et al., 2006). These same meta-analyses found that using theory to design an intervention had small positive effects (β = .10, β = .12)—a finding that was shared by 2 more moderately sized meta-analyses (Herbst et al., 2005; Johnson et al., 2003).

The information content of interventions had small positive effects in 4 of the 8 tests on condom use/unprotected sex. As shown in Online Resource 1, Cohen’s d effect sizes were between .09 and .40 larger when information was provided about the mechanisms of HIV, STI/HIV transmission or disease prevention methods. However, 3 of the 4 positive effects were based on meta-analyses that shared many of the same intervention studies (Albarracin et al., 2005; Albarracin et al., 2008; Durantini et al., 2006). There was also no conclusive evidence that including a motivational enhancement component within an intervention enhanced efficacy—although the inclusion of attitudinal arguments was found to have positive effects in around half of the meta-analyses where this moderator was tested. However, the inclusion of threat/fear-inducing or normative arguments may be just as likely to produce negative, rather than positive, effects. Although, there is some evidence that the use of fear might be effective with Latino groups (Albarracin et al., 2008) or within interventions conducted in groups, rather than at an individual or community level (Johnson et al., 2005). Although further research is needed to support these observations, these findings highlight how the effectiveness of some techniques might be dependent on specific population or intervention characteristics.

The most consistent moderator effects emerged for the skills components of the interventions. Although there was no evidence that interventions with a variety or mixture of skills training produced significant larger effect sizes, coding interventions according to more specific types of training such as training in condom skills, intrapersonal skills, and interpersonal skills, did show the potential value of these techniques. The effects were most consistent for condom skills and intrapersonal skills with 7 small to medium sized positive effects for each moderator across a range of different meta-analyses, including 3 of the 4 that focused on adolescent/youth populations (Johnson et al., 2003; Johnson et al., 2011; Scott-Sheldon et al., 2013).

## Discussion

A growing number of meta-analyses of STI/HIV prevention interventions have explored the sources of heterogeneity of effect sizes by testing the extent that various study characteristics moderate effect sizes. This meta-review synthesizes the results from 37 meta-analyses identified through a systematic search of the published literature. A range of mode of delivery, communicator and content dimensions were examined and consistent positive effects were found for a small number of characteristics including matching the gender or ethnicity of the communicator to the intervention recipients, group targeting or tailoring of the intervention, use of a theory to underpin intervention design, providing factual information, presenting arguments designed to change attitudes, and providing condom skills and intrapersonal skills training.

Although the use of theory moderator was not specific to a particular theory, our findings do lend support to the Information-Motivation and Behavioral Skills (IMB) model of HIV preventive behavior (Fisher & Fisher, 1992). This model proposes that information and behavioral skills are necessary, but not sufficient, for HIV prevention. People’s attitudes towards HIV prevention are also an important determinant of their motivation to initiate and maintain preventive behavior. The role of motivational enhancement and skills training was also highlighted in the meta-review conducted by Johnson et al. (2014), but the broader scope of our analysis has identified the potentially important roles of features such as matching the person delivering the intervention and targeting the content to the characteristics of the recipient. This highlights the value of designing and delivering interventions which are aimed at modifying IMB components in a group-appropriate fashion.

Also, by reporting the non-significant and negative effects alongside the positive effects, our meta-review highlights dimensions that either make no difference or could potentially compromise intervention efficacy. This includes dimensions that we might have expected to make a positive difference, such as the overall duration, number of sessions, peer delivery, tailoring to the individual, use of threat/fear induction methods, and normative arguments.

However, non-significant effects were quite prevalent and we need to be cautious about ruling out the potential value of some dimensions when in some meta-analyses the lack of significance might be attributable to lack of statistical power. We highlighted k < 20 as a small sample where lack of power might be an issue, although it should be noted that even with 20 studies the moderator effect size would need to be quite large to produce a significant effect. Meta-analyses probably need at least 50 or 60 studies to have sufficient power to detect even medium moderator effect sizes. Notably only 10 of the 37 meta-analyses included in this meta-review were based on 50 or more studies and only three of those included literature published within the last 5 years: LaCroix et al. (2014) k = 58; Scott-Sheldon et al. (2011) k = 67; and Tan et al. (2012) k = 52. Notably the largest reviews that include over 100 studies do not include any literature published within the last 10 years: Albarracin et al. (2005) k = 200; Albarracin et al. (2008) k = 350; Durantini et al. (2006) k = 166; Earl and Albarracin (2007) k = 180. This is probably because the most recent meta-analyses have tended to adopt increasingly restrictive inclusion criteria (i.e., focussing on particular types of interventions or population groups) which limit the potential to statistically examine moderators of intervention efficacy.

There are some limitations to this meta-review that need to be considered when interpreting the findings. Firstly, although we conducted a systematic and thorough search of the literature, we cannot rule out the possibility that relevant meta-analyses were not included. Secondly, we are reliant on the original authors’ literature search, data extraction, and analysis. Our synthesis relies not only on the thoroughness of the literature search and reliability of the coding of dimensions, but also the adequacy and accuracy of the statistical methods used to compute effect sizes and test moderator effects. Bearing in mind that all of the meta-analyses are published in peer reviewed journals we have placed some faith in the fact that the meta-analyses were conducted appropriately. However, there were some differences in the methods used to compute effect sizes (e.g., whether they were adjusted for baseline differences), and to test moderator effects (e.g., whether analyses were based on fixed, random, or mixed effects assumptions and use of Bonferroni corrected significance values), that may contribute towards the different patterns of results found between meta-analyses. There is also the possibility that we may have miscategorized the dimensions. Although the coding was checked between two researchers, the definitions used by some meta-analysts for their tested moderators were not always provided in detail. Also, some dimensions had quite broad definitions that may have picked up on subtly different issues. Group targeting/tailoring for example included both whether an intervention was targeted at a particular group and also whether the information was designed to be specific to the target audience. We grouped these two features together, but this could have masked different effects on intervention efficacy. Finally, the insights gained from this meta-review are somewhat restricted to identifying the moderators of intervention effect sizes for behavioral outcomes like condom use, rather than biomarker-confirmed outcomes such as STI/HIV infection rates. Our insights were restricted because only 7 of the 37 meta-analyses tested moderator effects on STI/HIV incidence. If we want to demonstrate the clinical relevance of behavioral interventions, there clearly needs to be more research which evaluates the effects on STI/HIV infection rates and considers their role relative to innovations in pharmacological prevention such as pre-exposure prophylaxis (Centers for Disease Control and Prevention, 2014).

Despite its limitations, this meta-review has advanced our understanding of factors linked to improved efficacy of behavioral interventions. It has also highlighted deficiencies in the existing meta-analytic literature including the tendency to narrow the focus and inclusion criteria. The narrow focus of many of the meta-analyses conducted in recent years has undermined the reliability of the moderator analyses that have been conducted. To further our understanding an up-to-date and less restricted meta-analysis of the HIV prevention literature is needed. A less restricted meta-analysis might also enable not only more rigorous multivariate tests of moderating factors but also an exploration of how the intervention delivery, communicator, and content factors interact with each other and other characteristics, like the study date, type of recipients, or country the study was conducted in. This could include testing some of the interactions tentatively highlighted in this meta-review, for example whether skills-based techniques work better with adolescents or threat/fear induction messages backfire when delivered to certain cultural groups. Exploring the role of factors like the study date would also provide an indication of whether the efficacy of behavioral interventions has changed over time. This type of analysis could provide insights into whether intervention efficacy has been influenced by innovations in the design of interventions or by changing external circumstances such as improved treatment or the broader social context.

The findings of this meta-review suggest that HIV/STI prevention interventions should involve a number of features. Researchers should consider who delivers the intervention, as interventions that match the gender or ethnicity of the communicator to the recipients tend to be more successful. In terms of content, there seems to be value in designing interventions that are group targeted or tailored, use theory to underpin intervention design, provide factual information, present arguments designed to change attitudes, and provide condom skills/intrapersonal skills training. In designing interventions, it is worth noting that the duration and number of sessions did not affect intervention success. Also, expert delivery was not more successful than peer delivery. These findings have important implications for the field and highlight how less labor-intensive (and thus cheaper) interventions may be as successful as those that are more labor-intensive. The specific method of delivery might however be important and a priority for future research is to compare traditional face-to-face approaches against novel methods which use social media and mHealth applications.

## Electronic supplementary material

Below is the link to the electronic supplementary material.

Supplementary material 1 (DOCX 59 kb)

Supplementary material 2 (DOCX 37 kb)
