# Supplementary material for: A synthesis of meta-analytic evidence of behavioral interventions to reduce HIV/STIs
Source: J Behav Med. 2016 Jan 30;39:371–85. doi: 10.1007/s10865-016-9714-1 (PMC4853449; doi:10.1007/s10865-016-9714-1)
Supplement: Supplementary file 1 — Supplementary material 1 (DOCX 59 kb) [file 10865_2016_9714_MOESM1_ESM.docx]

Online Resource 1.

Online supplementary material for *Covey, J., Rosenthal-Stott H.E.S., Howell, S. J. A synthesis of meta-analytic evidence of behavioral interventions to reduce HIV/STIs. Journal of Behavioral Medicine.

*Corresponding author: Department of Psychology, Durham University, Queen’s Campus, Stockton-on-Tees, TS17 6BH, UK. email: j.a.covey@durham.ac.uk

Significant and non-significant moderators of effect sizes for condom use/ unprotected sex

When moderators were significant the magnitude of the effect is shown by one of four measures depending on how the results of the moderator analysis were reported in the paper: β (standardised regression coefficient), r (correlation coefficient), and dΔ (difference between the Cohen’s d effect sizes at each level of the moderator). Effect sizes reported as odds-ratios were converted to Cohen’s d using the spreadsheet available at www.stat-help.com.

When moderators were non-significant (ns) the superscripts k and B indicate reduced power factors – (ns)^k^ when the number of studies used in the moderator analysis was fewer than 20 and (ns)^B^ when Bonferroni corrected significance levels were used.

| Mode of delivery dimensions | Duration | Session number | School setting | Clinic setting | Commun-ity setting | Group delivery |
| --- | --- | --- | --- | --- | --- | --- |
| (+) | 6 | 2 | 0 | 2 | 0 | 4 |
| (-) | 2 | 1 | 3 | 1 | 2 | 2 |
| ns (k<20, B)^[[1]](#endnote-1)^ | 15 (4,1) | 7 (3,0) | 4 (1,0) | 7 (3,0) | 6 (1,0) | 8 (3,0) |
| Albarracin D. et al. (2005) | (ns) | - | (ns) | (ns) | (ns) | (ns) |
| Albarracin D. et al. (2003) | (ns) | - | r=-.34 | - | - | - |
| Albarracin J. et al. (2008)  Low percent Latino  High percent Latino | -  - | -  - | -  - | d_Δ_=0.43^m^  d_Δ_=-0.12^m^ | d_Δ_=-0.18^m^  (ns) | d_Δ_=0.14^m^  (ns) |
| Chin et al. (2012) | (ns) | - | (ns) | - | (ns) | - |
| Crepaz et al. (2007) | (ns)^k^ | (ns)^k^ | - | (ns)^k^ | - | (ns)^k^ |
| Crepaz et al. (2009) | (ns) | (ns) | - | (ns) | (ns) | (ns) |
| Darbes et al. (2008) | (ns) | (ns) | - | (ns) | (ns) | (ns) |
| Durantini et al. (2006) | (ns) | - | β=-.23 | β=.44 | β=-.24 | β=.16 |
| Earl & Albarracin D. (2007) | - | - | - | - | - | - |
| Eaton et al. (2012)^^[[2]](#endnote-2)^^ | - | - | - | - | - | - |
| Henny et al. (2012) | (ns) | (ns) | - | - | - | - |
| Herbst et al. (2007) | - | d_Δ_=0.22 | - | (ns)^k^ | (ns)^k^ | (ns)^k^ |
| Herbst et al. (2005) | - | - | - | - | - | - |
| Higa et al. (2013) | (ns)^k^ | (ns)^k^ | - | - | - | (ns)^k^ |
| Huedo-Medina et al. (2010) | (ns)^B^ | - | β=-.33 | - | - | d_Δ_=-0.15 |
| Johnson B.T et al. (2003) | - | - | (ns) | - | - | (ns) |
| Johnson B.T et al. (2011) | - | (ns) | - | - | - | - |
| Johnson B.T. et al. (2009)  Short-term  Intermediate  Long-term | (ns)  β=.38^m^  β=.75^m^ | -  -  - | -  -  - | -  -  - | -  -  - | -  -  - |
| Johnson W.D. et al. (2005) | - | - | - | - | - | - |
| LaCroix et al. (2013) | (ns) | - | - | - | - | d_Δ_=-0.45 |
| LaCroix et al. (2014) | β=.48 | - | - | - | - | - |
| Lennon et al. (2012) | - | - | - | - | - | - |
| Liu et al. (2014) | - | - | - | - | - | - |
| Meader et al. (2013) | - | - | - | (ns) | - | - |
| Mullen et al. (2002)^[[3]](#endnote-3)^ | - | (ns)^k^ | (ns)^k^ | - | - | - |
| Neumann et al. (2002) | - | - | - | (ns)^k^ | - | (+)^[[4]](#endnote-4)^ |
| Noar et al. (2009) | - | d_Δ_=0.13 | - | - | - | - |
| Prendergast et al. (2001) | (ns)^k^ | - | - | - | - | - |
| Reid et al. (2014) | - | - | - | - | - | - |
| Scott-Sheldon et al. (2010)^[[5]](#endnote-5)^  Short-term  Intermediate  Long-term | β=.27  β=.40  β=.72 | -  -  - | -  -  - | -  -  - | -  -  - | -  -  - |
| Scott-Sheldon et al. (2011) | (ns) | - | - | - | - | - |
| Scott-Sheldon et al. (2013)^[[6]](#endnote-6)^ | β=-.54 | β=-.55 | - | - | - | - |
| Swanton et al. (2015) | (ns)^k^ | - | - | - | - | - |
| Tan et al. (2012)^[[7]](#endnote-7)^ | β=-.29 | - | - | - | - | d_Δ_=0.46 |
| Tyson et al. (2014) | - | - | - | - | - | - |
| Xiao et al. (2012) | - | - | - | - | - | - |
| Zheng & Zheng (2012) | - | - | - | - | - | - |

| Communicator dimensions | Peer delivery | Expert delivery | Matched ethnicity | Matched gender | Similar age |
| --- | --- | --- | --- | --- | --- |
| (+) | 4 | 6 | 6 | 8 | 3 |
| (-) | 3 | 2 | 1 | 0 | 0 |
| ns (k<20, B) | 4 (1,0) | 3 (0,0) | 7 (1,0) | 4 (0,0) | 5 (0,0) |
| Albarracin D. et al. (2005) | - | - | - | - | - |
| Albarracin D. et al. (2003) | - | - | - | - | - |
| Albarracin J. et al. (2008)  Low percent Latino  High percent Latino | -  - | d_Δ_=0.30^m^  (ns) | d_Δ_=0.21^m^  (ns) | d_Δ_=0.33^m^  (ns) | d_Δ_=0.12^m^  (ns) |
| Chin et al. (2012) | (ns) | - | - | - | - |
| Crepaz et al. (2007) | - | d_Δ_=-0.17 | d_Δ_=0.25 | - | - |
| Crepaz et al. (2009) | (ns) | - | (ns) | d_Δ_=0.38 | - |
| Darbes et al. (2008) | d_Δ_=0.25 | - | (ns) | - | - |
| Durantini et al. (2006)  Predominantly male  Predominantly female  Predominantly European  Predominantly African  Predominantly <21  Predominantly >21 | -  -  -  -  -  - | d_Δ_=0.17  d_Δ_=0.23  (ns)  d_Δ_=0.32  (ns)  d_Δ_=0.27 | d_Δ_=-0.12  d_Δ_=0.29  (ns)  d_Δ_=0.16  d_Δ_=0.21  d_Δ_=0.14 | (ns)  d_Δ_=0.36  d_Δ_=0.14  d_Δ_=0.32  d_Δ_=0.14  d_Δ_=0.28 | (ns)  (ns)  d_Δ_=0.13  (ns)  d_Δ_=0.23  (ns) |
| Earl & Albarracin D. (2007) ^^[[8]](#endnote-8)^^ | - | - | - | - | - |
| Eaton et al. (2012) | - | - | - | - | - |
| Henny et al. (2012) | - | - | (ns) | d_Δ_=0.32 | - |
| Herbst et al. (2007) | d_Δ_=-0.21 | - | (ns)^k^ | - | - |
| Herbst et al. (2005) | - | - | - | - | - |
| Higa et al. (2013) | (+)^[[9]](#endnote-9)^ | - | - | - | - |
| Huedo-Medina et al. (2010) | - | - | - | - | - |
| Johnson B.T et al. (2003) | (ns) | - | (ns) | (ns) | - |
| Johnson B.T et al. (2011) | - | - | - | - | - |
| Johnson B.T. et al. (2009) | - | - | - | - | - |
| Johnson W.D. et al. (2005) | - | - | - | - | - |
| LaCroix et al. (2013) | - | - | - | - | - |
| LaCroix et al. (2014) | - | - | - | - | - |
| Lennon et al. (2012) | - | - | - | - | - |
| Liu et al. (2014) | (ns)^k^ | - | - | - | - |
| Meader et al. (2013) | - | - | - | - | - |
| Mullen et al. (2002) | - | - | - | - | - |
| Neumann et al. (2002) | - | - | - | - | - |
| Noar et al. (2009) | - | - | - | - | - |
| Prendergast et al. (2001) | r=.67 | - | - | - | - |
| Reid et al. (2014) | - | - | - | - | - |
| Scott-Sheldon et al. (2010) | - | - | - | - | - |
| Scott-Sheldon et al. (2011) | - | - | (ns) | (ns) | - |
| Scott-Sheldon et al. (2013) | - | β=.31 | - | - | - |
| Swanton et al. (2015) | - | - | - | - | - |
| Tan et al. (2012) | - | - | - | - | - |
| Tyson et al. (2014) | - | - | - | - | - |
| Xiao et al. (2012) | d_Δ_=0.21 | d_Δ_=-0.21 | - | - | - |
| Zheng & Zheng (2012)  Most recent intercourse  Intercourse in last six months | d_Δ_=-0.24^^[[10]](#endnote-10)^^  d_Δ_=-0.20^[[11]](#endnote-11)^ | - | - | - | - |

| Content dimensions (1 of 3) | Group targeting/ tailoring | Individual tailoring | Formative research | Theory-based | Address barriers |
| --- | --- | --- | --- | --- | --- |
| (+) | 9 | 3 | 2 | 4 | 2 |
| (-) | 1 | 4 | 2 | 0 | 0 |
| ns (k<20, B) | 9 (2,0) | 7 (1, 1) | 4 (2,0) | 4 (2,0) | 2 (0,1) |
| Albarracin D. et al. (2005) | Ethnicity (ns) Gender β=.11 | - | β=-.12 | β=.10 | - |
| Albarracin D. et al. (2003) | - | - | r=.43 | - | - |
| Albarracin J. et al. (2008) | - | - | - | - | - |
| Chin et al. (2012) | (ns) | - | - | - | - |
| Crepaz et al. (2007) | (ns)^k^ | - | (ns)^k^ | - | - |
| Crepaz et al. (2009) | d_Δ_=0.28 | - | (ns) | - | - |
| Darbes et al. (2008) | (ns) | - | - | (ns) | - |
| Durantini et al. (2006) | Ethnicity (ns)  Gender β=.15 | - | β=-.08 | β=.12 | - |
| Earl & Albarracin D. (2007) | - | - | - | - | - |
| Eaton et al. (2012) | - | - | - | - | - |
| Henny et al. (2012) | (ns) | - | (ns) | - | - |
| Herbst et al. (2007) | - | - | (ns)^k^ | (ns)^k^ | d_Δ_=0.36 |
| Herbst et al. (2005) | - | - | - | d_Δ_=0.32 | - |
| Higa et al. (2013) | - | - | - | - | - |
| Huedo-Medina et al. (2010) | - | (ns)^B^ | - | - | (ns^B^) |
| Johnson B.T et al. (2003) | - | (ns) | - | (+)^[[12]](#endnote-12)^ | - |
| Johnson B.T et al. (2011) | - | (ns) | - | - | - |
| Johnson B.T. et al. (2009)  Short-term  Intermediate  Long-term | -  -  - | (ns)  β=.30  β=.33 | -  -  - | -  -  - | -  -  - |
| Johnson W.D. et al. (2005) | - | - | - | - | - |
| LaCroix et al. (2013) | - | - | - | - | - |
| LaCroix et al. (2014) | d_Δ_=0.25 | - | - | - | - |
| Lennon et al. (2012) | - | - | - | - | - |
| Liu et al. (2014) | - | - | - | - | - |
| Meader et al. (2013) | - | - | - | - | - |
| Mullen et al. (2002) | - | - | - | - | - |
| Neumann et al. (2002) | - | - | - | - | - |
| Noar et al. (2009) | - | d_Δ_=0.25 | - | (ns)^k^ | - |
| Prendergast et al. (2001) | - | - | - | - | - |
| Reid et al. (2014)  Whites negative attitude  Whites positive attitude | d_Δ_=0.34  (ns) | -  - | -  - | -  - | -  - |
| Scott-Sheldon et al. (2010)  Short-term  Intermediate  Long-term | β=.36^m^  β=.39  β=.82 | (ns)  β=-.34  β=-.49 | -  -  - | -  -  - | -  -  - |
| Scott-Sheldon et al. (2011) | Ethnicity β=.32  Gender (ns) | (ns) | - | - | β=.32^m^ |
| Scott-Sheldon et al. (2013) | - | - | - | - | - |
| Swanton et al. (2015) | - | - | - | - | - |
| Tan et al. (2012) | - | β=-.15 | - | - | - |
| Tyson et al. (2014) | - | - | - | - | (ns) |
| Xiao et al. (2012) | - | - | d_Δ_=0.36 | (ns) | - |
| Zheng & Zheng (2012)  Most recent intercourse  Intercourse in last six months | (ns)^k^  d_Δ_=-0.17^[[13]](#endnote-13)^ | (ns)^k^  d_Δ_=-0.09^[[14]](#endnote-14)^ | -  - | -  - | -  - |

| Content dimensions (2 of 3) | Information | Motivation enhance-ment | Threat/ fear induction | Attitudinal arguments | Normative arguments |
| --- | --- | --- | --- | --- | --- |
| (+) | 4 | 2 | 3 | 5 | 2 |
| (-) | 1 | 0 | 4 | 2 | 4 |
| ns (k<20, B) | 3 (0,0) | 9 (1,0) | 6 (3,1) | 4 (1,1) | 6 (1,0) |
| Albarracin D. et al. (2005)  Active interventions  Passive interventions | d_Δ_=0.17  (ns) | -  - | d_Δ_=-0.40  d_Δ_=-0.02 | d_Δ_=0.14  d_Δ_=0.10 | d_Δ_=-0.41  (ns) |
| Albarracin D. et al. (2003) | d_Δ_=-0.05 | - | (ns) | d_Δ_=0.22^m^ | (ns) |
| Albarracin J. et al. (2008)  Low percent Latino  High percent Latino | d_Δ_=0.09^m^  - | - | d_Δ_=-0.24^m^  d_Δ_=0.10^m^ | d_Δ_=0.05^m^  d_Δ_=-0.17^m^ | d_Δ_=-0.09^m^  d_Δ_=-0.79^m^ |
| Chin et al. (2012) | - | - | - | - | - |
| Crepaz et al. (2007) | - | - | (ns)^k^ | (ns)^k^ | - |
| Crepaz et al. (2009) | - | (ns) | - | - | (ns) |
| Darbes et al. (2008) | - | (ns) | - | (ns) | d_Δ_=0.38 |
| Durantini et al. (2006) | β=.03 | - | β=-.16 | β=-.02 | β=-.25 |
| Earl & Albarracin D. (2007) | - | - | d_Δ_=-0.43 | - | - |
| Eaton et al. (2012) | - | - | - | - | - |
| Henny et al. (2012) | (ns) | (ns) | - | (ns) | (ns) |
| Herbst et al. (2007) | - | - | - | - | d_Δ_=0.25 |
| Herbst et al. (2005) | - | - | - | - | - |
| Higa et al. (2013) | - | - | - | - | - |
| Huedo-Medina et al. (2010) | - | - | - | - | - |
| Johnson B.T et al. (2003) | - | - | - | - | - |
| Johnson B.T et al. (2011) | - | d_Δ_=0.34^m^ | - | - | - |
| Johnson B.T. et al. (2009) | - | - | - | - | - |
| Johnson W.D. et al. (2005)  Individual  Community  Group | -  -  - | -  -  - | (+)^^[[15]](#endnote-15)^^  (ns)^k^  (+)^[[16]](#endnote-16)^ | -  -  - | -  -  - |
| LaCroix et al. (2013) | - | - | - | - | - |
| LaCroix et al. (2014) | - | - | - | - | - |
| Lennon et al. (2012) | d_Δ_=0.40 | - | - | - | - |
| Liu et al. (2014) | - | - | - | - | - |
| Meader et al. (2013) | - | (ns) | - | - | - |
| Mullen et al. (2002) | - | - | (ns)^k^ | - | - |
| Neumann et al. (2002) | - | - | - | - | - |
| Noar et al. (2009) | - | - | - | - | - |
| Prendergast et al. (2001) | - | - | - | - | - |
| Reid et al. (2014) | - | - | - | - | - |
| Scott-Sheldon et al. (2010)  Short-term  Intermediate  Long-term | -  -  - | (ns)  (ns)  (ns)^k^ | -  -  - | -  -  - | -  -  - |
| Scott-Sheldon et al. (2011) | - | (ns) | - | - | - |
| Scott-Sheldon et al. (2013) | - | - | - | - | (ns)^k^ |
| Swanton et al. (2015) | - | - | - | - | - |
| Tan et al. (2012) | - | β=.27 | (ns)^B^ | (ns)^B^ | - |
| Tyson et al. (2014) | (ns) | (ns) | - | d_Δ_=0.39 | (ns) |
| Xiao et al. (2012) | - | - | - | - | - |
| Zheng & Zheng (2012) | - | - | - | - | - |

| Content dimensions (3 of 3) | Address self-efficacy | Behavioral skills arguments | Skills (mixed) | Condom skills | Intra-personal skills | Inter-personal skills |
| --- | --- | --- | --- | --- | --- | --- |
| (+) | 1 | 5 | 0 | 7 | 7 | 5 |
| (-) | 0 | 1 | 0 | 1 | 2 | 2 |
| ns (k<20, B) | 3 (2,0) | 0 | 8 (4,0) | 7 (2,1) | 7 (2,0) | 9 (3,1) |
| Albarracin D. et al. (2005)  Active interventions  Passive interventions | - | d_Δ_=0.18  d_Δ_=0.16 | -  - | (ns)  - | d_Δ_=0.41  - | d_Δ_=-0.11  - |
| Albarracin D. et al. (2003) | - | d_Δ_=0.15^m^ | - | - | - | - |
| Albarracin J. et al. (2008)  Low percent Latino  High percent Latino | -  - | d_Δ_=0.30^m^  d_Δ_=-0.25^m^ | -  - | d_Δ_=0.04^m^  d_Δ_=-0.29^m^ | d_Δ_=0.36^m^  d_Δ_=-0.36^m^ | (ns)  d_Δ_=-0.46^m^ |
| Chin et al. (2012) | - | - | - | - | - | - |
| Crepaz et al. (2007) | (ns)^k^ | - | - | (ns)^k^ | (ns)^k^ | (ns)^k^ |
| Crepaz et al. (2009) | d_Δ_=0.28 | - | - | d_Δ_=0.39 | - | - |
| Darbes et al. (2008) | (ns) | - | (ns) | - | - | - |
| Durantini et al. (2006) | - | β=.22 | - | β=.20 | β=.23 | β=.14 |
| Earl & Albarracin D. (2007) | - | - | - | - | - | - |
| Eaton et al. (2012) | - | - | - | - | - | - |
| Henny et al. (2012) | - | - | - | (ns) | (ns) | (ns) |
| Herbst et al. (2007) | (ns)^k^ | - | - | (ns)^k^ | d_Δ_=0.41 | (ns)^k^ |
| Herbst et al. (2005) | - | - | - | - | - | - |
| Higa et al. (2013) | - | - | - | - | - | (+)^[[17]](#endnote-17)^ |
| Huedo-Medina et al. (2010) | - | - | - | - | - | (ns)^B^ |
| Johnson B.T et al. (2003) | - | - | - | d_Δ_=0.35^m^ | - | - |
| Johnson B.T et al. (2011) | - | - | - | d_Δ_=0.34^m^ | - | (ns) |
| Johnson B.T. et al. (2009)  Short-term  Intermediate  Long-term | -  -  - | -  -  - | -  -  - | -  -  - | β=.25^m^  (ns)  (ns) | (ns)  β=.20  β=.39 |
| Johnson W.D. et al. (2005)  Individual  Community  Group | -  -  - | -  -  - | -  -  - | -  -  - | (ns)  (+)^^[[18]](#endnote-18)^^  (ns) | -  -  - |
| LaCroix et al. (2013) | - | - | - | d_Δ_=-0.18^[[19]](#endnote-19)^ | - | - |
| LaCroix et al. (2014) | - | - | - | - | - | - |
| Lennon et al. (2012) | - | - | - | - | - | - |
| Liu et al. (2014) | - | - | - | - | - | - |
| Meader et al. (2013) | - | - | - | (ns) | - | - |
| Mullen et al. (2002) | - | - | - | - | (ns)^k^ | (ns)^k^ |
| Neumann et al. (2002) | - | - | (ns)^k^ | - | - | - |
| Noar et al. (2009) | - | - | (ns)^k^ | - | - | - |
| Prendergast et al. (2001) | - | - | (ns)^k^ | - | r=.59 | - |
| Reid et al. (2014) | - | - | - | - | - | - |
| Scott-Sheldon et al. (2010)  Short-term  Intermediate  Long-term | -  -  - | -  -  - | (ns)  (ns)  (ns)^k^ | -  -  - | -  -  - | -  -  - |
| Scott-Sheldon et al. (2011) | - | - | - | (ns) | β=-.28 | (ns) |
| Scott-Sheldon et al. (2013) | - | - | - | β=.30 | - | - |
| Swanton et al. (2015) | - | - | - | - | - | - |
| Tan et al. (2012) | - | - | - | (ns)^B^ | - | (+)^[[20]](#endnote-20)^ |
| Tyson et al. (2014) | - | - | (ns) | - | - | - |
| Xiao et al. (2012) | - | - | - | - | - | - |
| Zheng & Zheng (2012) | - | - | - | - | - | - |

1. Number of non-significant moderator effects with reduced power factors - k<20 and Bonferroni corrected significance levels [↑](#endnote-ref-1)
2. Moderator analysis were only conducted on the effect sizes for STI rates [↑](#endnote-ref-2)
3. Although the authors adopted Bonferroni corrected significance levels (p=.004) all the p–values for non-significant moderator effects reported in this table (ns) were greater than the less conservative uncorrected significance level of p=.05 that was used in the majority of the meta-analyses reported in this paper [↑](#endnote-ref-3)
4. Larger effect sizes in interventions delivered to groups rather than individuals. No effect size data provided. [↑](#endnote-ref-4)
5. Although the authors adopted Bonferroni corrected significance levels (p=.004) the significance of the univariate effects reported in this table are based on the less conservative uncorrected significance level of p=.05 that was used in the majority of the meta-analyses reported in this paper. [↑](#endnote-ref-5)
6. Although the authors adopted Bonferroni corrected significance levels (p=.005) the significance of the univariate effects reported in this table are based on the less conservative uncorrected significance level of p=.05 that was used in the majority of the meta-analyses reported in this paper. The corrected significance level does however still apply to the multivariate tests in which only moderators that were found to be significant in univariate analysis (p<.005) were tested in a multivariate model. [↑](#endnote-ref-6)
7. The authors did not report the actual p-values for the non-significant effects and it should be noted that they adopted a much more conservative Bonferroni corrected significance level (p=.001) compared to the value of p=.05 used in the majority of the meta-analyses reported in this paper. This corrected significance level was also applied to the multivariate tests conducted for moderators that were found to be significant (p<.001) in the univariate analysis. [↑](#endnote-ref-7)
8. All coded strategies were entered simultaneously into a multivariate analysis [↑](#endnote-ref-8)
9. Fisher’s exact test showed that evidence-based interventions (EBIs) were significantly more likely to be delivered by peers than non-evidence based interventions (no-EBIs) (p<.05). No effect size data provided. [↑](#endnote-ref-9)
10. Smaller effect sizes in interventions which included peer-led education compared to interventions which used the popular opinion leader strategy [↑](#endnote-ref-10)
11. Smaller effect sizes in interventions which included peer-led education compared to interventions which used the popular opinion leader strategy [↑](#endnote-ref-11)
12. The use of behavioral theory was positively related to the success of the intervention but reduced to non-significance when other factors were controlled in the analysis. No effect size data reported. [↑](#endnote-ref-12)
13. Effect size smaller in group targeted interventions than mixed type interventions [↑](#endnote-ref-13)
14. Effect size smaller in individually tailored interventions than mixed type interventions [↑](#endnote-ref-14)
15. After controlling for other characteristics interventions which addressed perception of risk or losses rather than gains produced more favorable effects. No effect size data provided. [↑](#endnote-ref-15)
16. After controlling for other characteristics interventions which addressed perception of risk or losses rather than gains produced more favorable effects. No effect size data provided. [↑](#endnote-ref-16)
17. Fisher’s exact test showed that evidence-based interventions (EBIs) were significantly more likely to include content on sexual communication/ negotiation than non-evidence based interventions (no-EBIs) (p<.05). No effect size data provided. [↑](#endnote-ref-17)
18. After controlling for other characteristics interventions which promoted personal skills produced more favourable effects. No effect size data provided. [↑](#endnote-ref-18)
19. Effect sizes greater when intervention included active rather than passive condom skills training [↑](#endnote-ref-19)
20. Interpersonal skills training was related to condom use efficacy on a bivariate basis but was not significant in the multivariate model. Effect size not reported. [↑](#endnote-ref-20)
