# Supplementary material for: A synthesis of meta-analytic evidence of behavioral interventions to reduce HIV/STIs
Source: J Behav Med. 2016 Jan 30;39:371–85. doi: 10.1007/s10865-016-9714-1 (PMC4853449; doi:10.1007/s10865-016-9714-1)
Supplement: Supplementary file 2 — Supplementary material 2 (DOCX 37 kb) [file 10865_2016_9714_MOESM2_ESM.docx]

Online Resource 2.

Online supplementary material for *Covey, J., Rosenthal-Stott H.E.S., Howell, S. J. A synthesis of meta-analytic evidence of behavioral interventions to reduce HIV/STIs. Journal of Behavioral Medicine.

*Corresponding author: Department of Psychology, Durham University, Queen’s Campus, Stockton-on-Tees, TS17 6BH, UK. email: j.a.covey@durham.ac.uk

Significant and non-significant moderators of effect sizes for STI/HIV incidence

When moderators were significant the magnitude of the effect is shown by one of four measures depending on how the results of the moderator analysis were reported in the paper: β (standardised regression coefficient), r (correlation coefficient), and dΔ (difference between the Cohen’s d effect sizes at each level of the moderator). Effect sizes reported as odds-ratios were converted to Cohen’s d using the spreadsheet available at [www.stat-help.com](http://www.stat-help.com).

When moderators were not significant (ns) the superscripts k and B are used to indicate reduced power factors – (ns)^k^ when the number of studies used in the moderator analysis was fewer than 20 and (ns)^B^ when Bonferroni corrected significance levels were used.

| Mode of delivery dimensions | Duration | Session number | School setting | Clinic setting | Commun-ity setting | Group delivery |
| --- | --- | --- | --- | --- | --- | --- |
| (+) | 2 | 0 | 0 | 0 | 0 | 0 |
| (-) | 1 | 0 | 0 | 0 | 0 | 0 |
| ns (k<20, B) | 8 (4,0) | 3 (3,0) | 1 (1,0) | 3 (3,0) | 3 (3,0) | 3 (2,0) |
| Chin et al. (2012) | (ns)^k^ | - | (ns)^k^ | - | (ns)^k^ | - |
| Crepaz et al. (2007) | (ns)^k^ | (ns)^k^ | - | (ns)^k^ | - | - |
| Crepaz et al. (2009) | d_Δ_=1.61 | (ns)^k^ | - | (ns)^k^ | (ns)^k^ | (ns)^k^ |
| Darbes et al. (2008) | (ns)^k^ | (ns)^k^ | - | (ns)^k^ | (ns)^k^ | (ns)^k^ |
| Eaton et al. (2012) | d_Δ_=0.20 | - | - | - | - | - |
| Scott-Sheldon et al. (2010)^[[1]](#endnote-1)^  Short-term  Intermediate  Long-term | β=-74  (ns)  (ns) | -  -  - | -  -  - | -  -  - | -  -  - | -  -  - |
| Scott-Sheldon et al. (2011)  STI incidence  HIV incidence | (ns)  (ns)^k^ | -  - | -  - | -  - | -  - | -  - |
| Tan et al. (2012)^[[2]](#endnote-2)^ | (ns) | - | - | - | - | (ns) |

| Communicator dimensions | Peer delivery | Expert delivery | Matched ethnicity | Matched gender | Similar age |
| --- | --- | --- | --- | --- | --- |
| (+) | 1 | 0 | 1 | 0 | - |
| (-) | 0 | 0 | 0 | 0 | - |
| ns (k<20, B) | 2 (2,0) | 1 (1,0) | 3 (2,0) | 3 (2,0) | - |
| Chin et al. (2012) | (ns)^k^ | - | - | - | - |
| Crepaz et al. (2007) | - | (ns)^k^ | d_Δ_=0.32 | - | - |
| Crepaz et al. (2009) | d_Δ_=1.62 | - | (ns)^k^ | (ns)^k^ | - |
| Darbes et al. (2008) | (ns)^k^ | - | (ns)^k^ | - | - |
| Eaton et al. (2012) | - | - | - | - | - |
| Scott-Sheldon et al. (2010) | - | - | - | - | - |
| Scott-Sheldon et al. (2011)  STI incidence  HIV incidence | -  - | -  - | (ns)  - | (ns)  (ns)^k^ | -  - |
| Tan et al. (2012) | - | - | - | - | - |

| Content dimensions (1 of 3) | Information | Group targeting/ tailoring | Individual tailoring | Formative research | Theory-based |
| --- | --- | --- | --- | --- | --- |
| (+) | - | 1 | 0 | 1 | 1 |
| (-) | - | 0 | 0 | 0 | 0 |
| ns (k<20, B) | - | 8 (4,0) | 5 (1,0) | 1 (1,0) | 1 (1,0) |
| Chin et al. (2012) | - | (ns)^k^ | - | - | - |
| Crepaz et al. (2007) | - | (ns)^k^ | - | (ns)^k^ | d_Δ_=0.34 |
| Crepaz et al. (2009) | - | (ns)^k^ | - | d_Δ_=0.27 | - |
| Darbes et al. (2008) | - | (ns)^k^ | - | - | (ns)^k^ |
| Eaton et al. (2012) | - | - | - | - | - |
| Scott-Sheldon et al. (2010)  Short-term  Intermediate  Long-term | -  -  - | -  (ns)  (ns) | β=-.70  (ns)  (ns) | -  -  - | -  -  - |
| Scott-Sheldon et al. (2011)  STI incidence  HIV incidence | -  - | Cultural (ns)  Gender (ns)  Gender β=.45 | (ns)  (ns)^k^ | -  - | -  - |
| Tan et al. (2012) | - | - | (ns) | - | - |

| Content dimensions (2 of 3) | Motivation  enhancement | Threat/ fear induction | Attitudinal arguments | Normative arguments | Address barriers |
| --- | --- | --- | --- | --- | --- |
| (+) | 2 | 1 | 0 | 0 | 0 |
| (-) | 1 | 0 | 0 | 0 | 0 |
| ns (k<20, B) | 5 (2,0) | 1 (1,0) | 3 (2,0) | 2 (2,0) | 2 (1,0) |
| Chin et al. (2012) | - | - | - | - | - |
| Crepaz et al. (2007) | - | d_Δ_=-0.03^k^ | d_Δ_=-0.12^k^ | - | - |
| Crepaz et al. (2009) | (ns)^k^ | - | - | (ns)^k^ | - |
| Darbes et al. (2008) | (ns)^k^ | - | (ns)^k^ | (ns)^k^ | - |
| Eaton et al. (2012) | - | - | - | - | - |
| Scott-Sheldon et al. (2010)  Short-term  Intermediate  Long-term | β=-.70  β=.40^m^  (ns) | -  -  - | -  -  - | -  -  - | -  -  - |
| Scott-Sheldon et al. (2011)  STI incidence  HIV incidence | (ns)  β=.43 | -  - | -  - | -  - | (ns)  (ns)^k^ |
| Tan et al. (2012) | (ns) | β=.21 | (ns) | - | - |

| Content dimensions (3 of 3) | Address self-efficacy | Behavio-ral skills arguments | Skills (mixed) | Condom skills | Intra-personal skills | Inter-personal skills |
| --- | --- | --- | --- | --- | --- | --- |
| (+) | 1 | - | 0 | 1 | 0 | 1 |
| (-) | 0 | - | 1 | 0 | 1 | 0 |
| ns (k<20, B) | 2 (2,0) | - | 3 (1,0) | 4 (2,0) | 2 (1,0) | 3 (1,0) |
| Chin et al. (2012) | - | - | - | - | - | - |
| Crepaz et al. (2007) | (ns)^k^ | - | - | (ns)^k^ | (ns)^k^ | (ns)^k^ |
| Crepaz et al. (2009) | d_Δ_=0.34 | - | - | (ns)^k^ | - | - |
| Darbes et al. (2008) | (ns)^k^ | - | (ns)^k^ | - | - | - |
| Eaton et al. (2012) | - | - | - | - | - | - |
| Scott-Sheldon et al. (2010)  Short-term  Intermediate  Long-term | -  -  - | -  -  - | β=-.52  (ns)  (ns) | -  -  - | -  -  - | -  -  - |
| Scott-Sheldon et al. (2011)  STI incidence  HIV incidence | -  - | -  - | -  - | (ns)  β=.51 | β=-.35^m^  (ns) | (ns)  β=.43 |
| Tan et al. (2012) | - | - | - | (ns) | - | (ns) |

1. Although the authors adopted Bonferroni corrected significance levels (p=.004) the significance of the univariate effects reported in this table are based on the less conservative uncorrected significance level of p=.05 that was used in the majority of the meta-analyses reported in this paper. [↑](#endnote-ref-1)
2. The authors did not report the actual p-values for the non-significant effects and it should be noted that they adopted a much more conservative Bonferroni corrected significance level (p=.001) compared to the value of p=.05 used in the majority of the meta-analyses reported in this paper. This corrected significance level was also applied to the multivariate tests conducted for moderators that were found to be significant (p<.001) in the univariate analysis. [↑](#endnote-ref-2)
